# Variable reactive hyperemia in normotensive strains of rat

**DOI:** 10.14814/phy2.12052

**Published:** 2014-06-24

**Authors:** J. Brett Heimlich, David M. Pollock

**Affiliations:** 1 Department of Physiology, Georgia Regents University, Augusta, Georgia, USA; 2 Cardio‐Renal Physiology and Medicine, Division of Nephrology, Department of Medicine, The University of Alabama at Birmingham, Birmingham, Alabama, USA

**Keywords:** Flow‐mediated dilation, nitric oxide, reactive hyperemia

## Abstract

Previous studies from our laboratory report variation in nitric oxide (NO)‐dependent arterial pressure within the same strain of normotensive Sprague–Dawley rat dependent upon the commercial vendor supplying the rats. Clinical assessment of endothelial NO activity and endothelial function in general has used postocclusion, flow‐mediated dilation (FMD). Therefore, this study was conducted to determine whether the reactive hyperemic response was different between two normotensive strains from two different suppliers, Sprague–Dawley (SD) and Wistar–Kyoto (WKY) rats from Charles River (CR) and Harlan Laboratories (H), respectively. Rats were anesthetized and the femoral artery was occluded for 5 min, with femoral blood flow measured continuously by use of an ultrasonic perivascular flow probe. The average area under the reactive hyperemic response curve (3‐min duration) was not different between SD rats from CR (80 ± 23 mL/min∙s; *n* = 6) and H (94 ± 16 mL/min∙s; *n* = 6). As previously reported, blood pressures were higher in the SD rats from H versus CR. WKY rats from both suppliers had significantly larger hyperemia; 371 ± 67 versus 281 ± 71 mL/min∙s (*n* = 5) for the CR and H WKY rats, respectively, but again, were not different between vendors. Blood pressures in WKY rats were similar between vendors. These results suggest that differences in NO bioactivity are not discernable with an adapted FMD protocol in the rat and that normotensive strains of rat can have large differences in reactive hyperemia despite having similar blood pressures.

## Abstract (precis)

e12052

Much of the scientific literature presumes that the physiology of so‐called normal rat strains is identical even when obtained from different suppliers. We demonstrate here that the blood flow response in the hindlimb of the rat to acute ischemia shows consistency within a given strain, but vary tremendously from different strains of rat even though both are considered “normal” control strains, the Sprague–Dawley and Wistar–Kyoto rats.

## Introduction

Endothelial dysfunction is evident in multiple disease processes including chronic hyperglycemia associated with diabetes, hypertension, and hypercholesterolemia (Landmesser et al. 2000; Wong et al. 2010; Paneni et al. 2013). Endothelial dysfunction is also thought to contribute to increased resistance to blood flow, but also precedes the formation of atherosclerotic plaques in vascular disease (Gimbrone and Garcia‐Cardena 2013). Normal endothelial function relies on nitric oxide signaling and other endothelial‐derived relaxing factors communicating between endothelial and smooth muscle cells to produce smooth muscle relaxation and resulting decreased resistance to blood flow, a critical task of the vascular endothelium (Tousoulis et al. 2012).

Given the prominent role of endothelial function in pathologic processes, a reliable, noninvasive indicator of endothelial function would provide significant clinical utility in the diagnosis and treatment of cardiovascular disease in humans. The knowledge that vasodilation is dependent upon an intact endothelium and could be augmented by shear forces culminated in the assessment of flow‐mediated dilation (FMD) as a technique that would allow clinicians and scientists to evaluate endothelial function noninvasively (Furchgott and Zawadzki 1980; Pohl et al. 1986; Rubanyi et al. 1986; Celermajer et al. 1992). Thus, reactive hyperemia is used as a surrogate for FMD and is used in humans as a predictor of endothelial function. Furthermore, FMD assessment is also used as a relative predictor of nitric oxide (NO) bioavailability as the reactive hyperemic response is thought to be mediated via the buildup of metabolic byproducts and release of NO in a shear‐dependent fashion (Joannides et al. 1995; Green 2005). More recently, this technique has been adapted to test changes in endothelial function as a consequence of a stimulus or intervention (Neunteufl et al. 2000; Harris et al. 2008).

Considerable effort has been aimed at elucidating the role of NO in physiological and pathophysiological endothelial function in the past 20 or so years and much of this research has been conducted in animal models of various kinds, including the rat. Subsequently, variations within different strains of rat have been documented throughout the literature and are utilized to inform future research. Several studies relate strain differences in the functional response to pharmacological inhibition of NO synthase either through N*ω*‐Nitro‐L‐arginine methyl ester hydrochloride (L‐NAME) or N*ω*‐nitro‐L‐arginine (L‐NNA) administration, suggesting variation in NO‐dependent vascular resistance under otherwise normal conditions (Kawakami et al. 1999). Previous studies from our laboratory even note a dependence on the vendor supplying Sprague–Dawley rats in L‐NAME‐mediated hypertension (Pollock and Rekito 1998). The purpose of this study was to adapt the clinical FMD methodology to assay NO bioavailability in the rat and subsequently, determine whether the degree of reactive hyperemia (FMD) is dependent upon the strain of rat.

## Methods

### Animals

Male, 12‐ to 14‐week‐old Sprague–Dawley (SD) and Wistar–Kyoto (WKY) rats were obtained from both Harlan (H, Indianapolis, IN) and Charles River (CR, Wilmington, MA). Animals were maintained in accordance with the National Institutes of Health *Guide for the Care and use of Laboratory Animals* and approved and monitored by the Georgia Regents University Institutional Animal Care and Use Committee. Animals were housed under conditions of controlled temperature and humidity and exposed to a 12:12‐h light/dark cycle. All rats were given free access to chow and tap water.

### Blood flow and blood pressure measurements

Rats were weighed and anesthetized using thiobutabarbital (100 mg/kg) and maintained on a servo‐controlled heated surgery table at 37.0°C. A polyethylene (PE 205) tube was placed in the trachea to ensure a patent airway throughout the procedure. The left femoral artery was isolated and cannulated with PE 50 tubing to monitor blood pressure using a MacLab data acquisition system (AD Instruments, Milford, MA). The contralateral femoral artery was isolated and an ultrasonic Doppler flow probe (Transonic Systems Inc., Ithaca, NY) was secured in situ. Animals were allowed to stabilize 30 min before starting the reactive hyperemia clamping procedure. Femoral artery blood flow was recorded for 10 min as a baseline measurement and then clamped for 5 min using a 6x1 mm microserrefines vascular clamp (Fine Science Tools, Foster City, CA). Responses were recorded continuously after clamp release (time zero) for at least 20 min before repeating the next clamp procedure. The clamping procedure was repeated four times on the same limb in each animal. Area under the blood flow response curve above baseline was calculated using GraphPad Prism software (San Diego, CA) for 3 min post clamp and reactive hyperemic responses from the four clamping procedures were averaged for each animal. Reactive hyperemia responses were then grouped and averaged according to appropriate strain and supplier. Premyogenic assessment includes blood flow from time 0 through 10 sec while postmyogenic includes data between 11 and 100 sec.

### Statistics

A two‐way ANOVA was used with Bonferroni post hoc tests to compare individual means between groups. Linear regression analysis was used to determine the potential relationship between independent factors. Results are expressed as means ± SEM, with *P *<**0.05 being considered statistically significant.

## Results

Baseline values for mean arterial pressure, steady‐state blood flow in femoral arteries, body mass, and femoral artery vascular resistance are presented in Table 1. Mean arterial pressure (MAP) varied significantly depending on both strain and supplier. MAP was significantly higher in SD rats compared to WKY with post hoc Bonferroni tests determining Harlan SD rats have significantly increased MAP over Harlan WKY animals. There was also a significant interaction between strain and supplier on MAP. Femoral artery blood flow per 100 g body mass was significantly elevated in SD rats compared to WKY. Furthermore, post hoc analysis revealed that blood flow per 100 g body mass was also significantly greater in the H SD compared to H WKY. Body mass varied significantly as a result of both strain and supplier. H SD rats weighed significantly more than H WKY rats but CR SD rats were significantly lighter than their CR WKY counterparts. Femoral artery vascular resistance was calculated by dividing MAP by the baseline blood flow. There were no detectable differences based on strain or supplier in our analysis.

**Table 1.** Baseline characteristics of SD and WKY rats from Harlan and Charles River (CR).

	Blood pressure (mmHg)	Baseline blood flow (mL/min/100 g)	Body mass (g)	Vascular resistance (mmHg*min/mL)
Harlan SD	121 ± 4***	0.67 ± 0.09*	335 ± 10†	61.0 ± 9.9
Harlan WKY	94 ± 3	0.43 ± 0.03	261 ± 3	84.9 ± 5.9
CR SD	97 ± 5	0.48 ± 0.04	295 ± 9**	70.6 ± 5.6
CR WKY	90 ± 6	0.40 ± 0.04	338 ± 5	69.2 ± 7.9
*P*_Strain_	0.001	0.01	0.04	0.15
*P*_Supplier_	0.006	0.08	0.01	0.68
P_*Supplier_	0.04	0.20	0.0001	0.11

All data are mean ± SEM.

***P <**0.001 versus Harlan WKY; **P <**0.01 versus CR WKY; *P <**0.05 versus Harlan WKY; ^†^*P *<**0.001 versus Harlan WKY.

**Figure 1.**
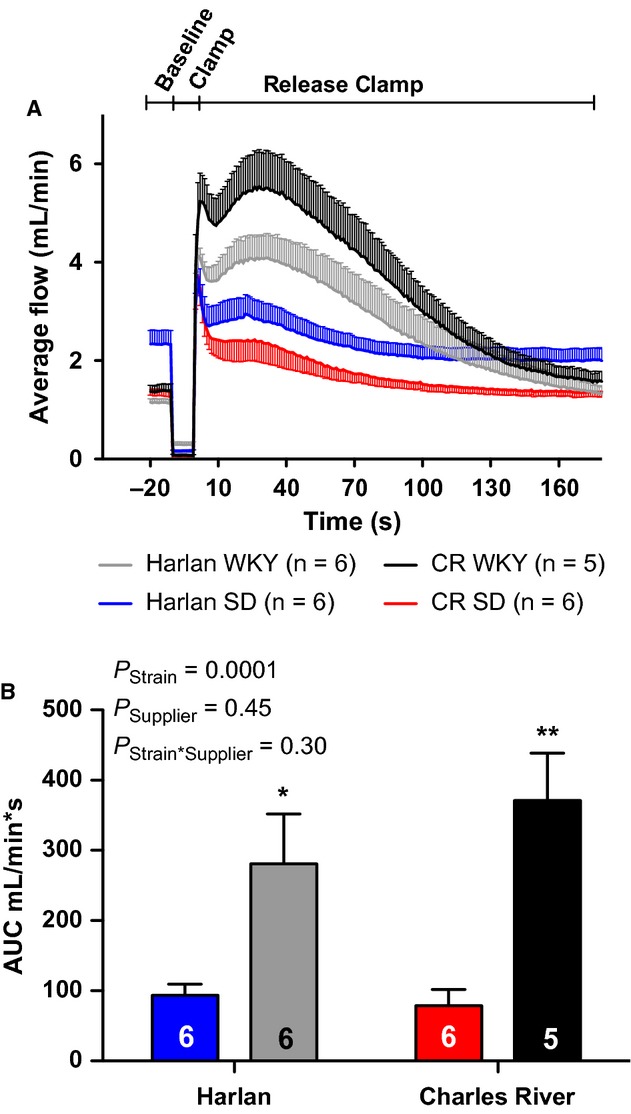
Average blood flow response to 5 min of femoral artery occlusion (A) and quantitation of total reactive hyperemia response (B) in SD and WKY rats from Harlan (H) and Charles River (CR) Laboratories. All data are mean ± SEM. *P <**0.05 versus Harlan SD; **P <**0.01 versus Harlan WKY.

**Figure 2.**
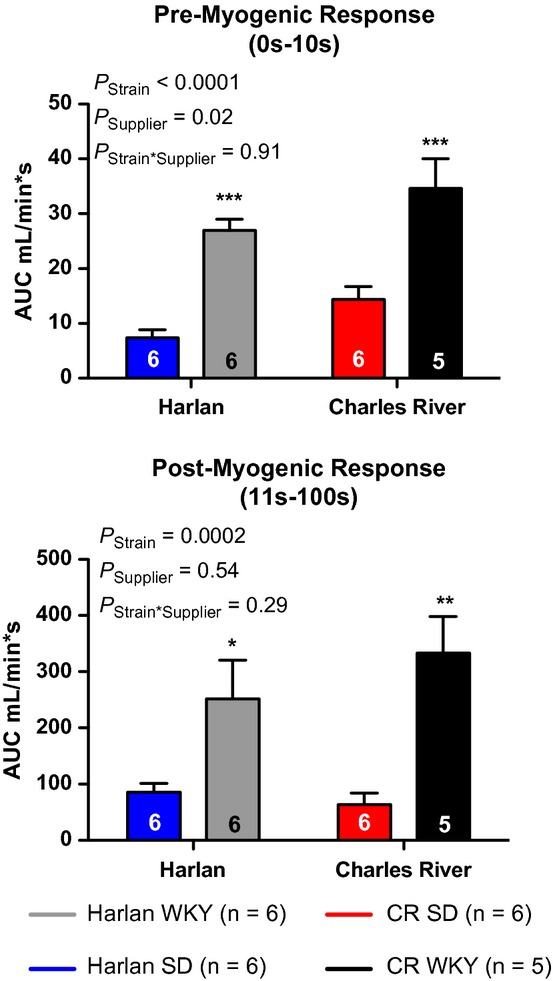
Premyogenic (A) and postmyogenic (B) reactive hyperemia responses in SD and WKY rats from Harlan (H) and Charles River (CR) Laboratories. *P <**0.05 versus Harlan SD; **P <**0.01 versus Harlan WKY; ***P <**0.001 versus animals of the opposite strain from the same distributor.

**Figure 3.**
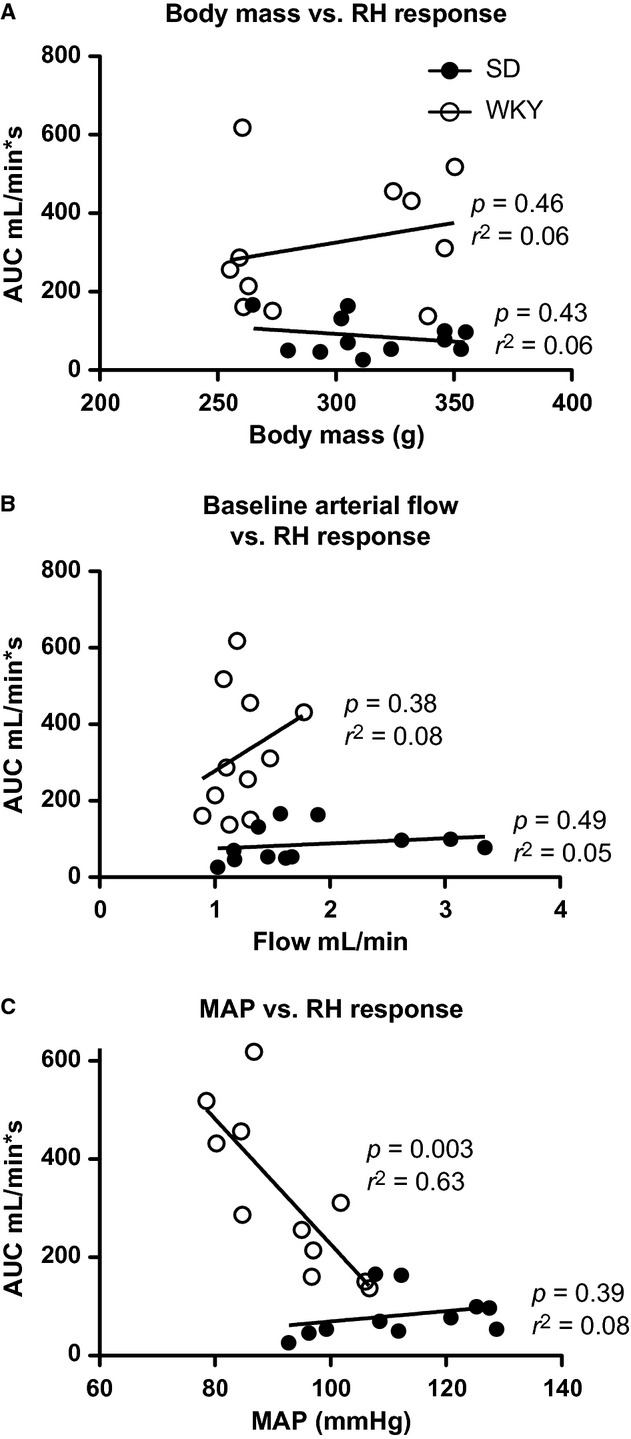
Linear regression plots for baseline parameters including body mass (A) and femoral artery blood flow (B) and mean arterial pressure (C).

Rats were subjected to femoral artery clamping and a summary tracing is given in Figure 1A. In response to 5‐min femoral artery clamping, WKY rats had a significantly more robust reactive hyperemia compared to SD rats that was independent of distributor (Fig. 1B). Clamp removal produces a sharp increase in blood flow and elicits a myogenic stretch reflex that initially limits blood flow until this response is overwhelmed by the release of vasoactive factors. This is illustrated in Figure 1A where an initial spike in blood flow immediately after clamp release is followed by a modest decrease reaching a nadir at 10 sec postclamp removal. This is termed premyogenic response. The increase seen in overall reactive hyperemia response was recapitulated in both the premyogenic and postmyogenic phases of reactive hyperemia, although to a lesser extent in the postmyogenic measurement (Fig. 2A and B). Additionally, a significant effect of distributor was also found in the premyogenic assessment (Fig. 2A). Noting significant variations in the average body mass, femoral artery blood flow, and MAP of animals at baseline, we plotted these parameters against the average reactive hyperemia response to clamping. We found no significant correlation between rat body mass or rat baseline femoral artery blood flow and the reactive hyperemia response (Fig. 3A and B) but found a moderate linear correlation between MAP and the AUC response that was dependent upon rat strain. As steady‐state blood pressure increased in WKY rats, a less robust reactive hyperemia response was observed, whereas no significant correlation could be found in SD animals (Fig. 3C).

## Discussion

Given the use of rats from a variety of breeding stocks, it is important to recognize and understand the underlying physiological differences among even normotensive, otherwise control, strains of rats. Our experiments were conducted with two very widely used strains of rat to demonstrate a dependence upon rat strain in the femoral artery reactive hyperemia response, with WKY rats having a larger reactive hyperemia response compared to SD rats. These results suggest that stimulated levels of NO bioavailability are variable given the rat strain, but are relatively constant between distributors since we did not see any difference in rats obtained from Harlan versus Charles River. Premyogenic and postmyogenic response components of the hyperemic response were observed in all groups of rat and the magnitude of these responses was again different between SD and WKY rats.

The WKY group had a larger overall hyperemic response, relative premyogenic responses, and accordingly higher postmyogenic blood flow regardless of distributor. WKY rats were originally derived as controls for the widely used spontaneously hypertensive rat (SHR) in 1963 and maintained as an outbred rat strain since 1971 (Kurtz and Morris 1987). Sprague–Dawley rats were first produced by Harlan Laboratories in 1925 and are widely available and outbred. Typical breeding strategies at both Harlan and Charles River have adopted random mating practices to limit the effects of genetic drift in their rat colonies. Prior selective breeding practices and incomplete inbreeding in the WKY strain could have potentially contributed to genetic alterations over time (Kurtz and Morris 1987; Pollock and Rekito 1998). However, since distributor specificity does not appear to affect the reactive hyperemia response, differences are likely attributable to inherent genetic differences between rat strains.

Analysis of previously published data using telemetry to study blood pressure data suggests that WKY rats have a greater resistance to hypertensive stimuli such as chronic angiotensin II infusion (Loria et al. 2010). This has led us to speculate that WKY rats have a greater capacity for opposing vasoconstrictor stimuli, which is consistent with our current observations that WKY rats have a more robust FMD response and suggests factors such as NO may be more active in WKY compared to SD rats.

Our findings in SD rats from different suppliers was somewhat surprising given our previously published studies that Harlan rats had a much larger hypertensive response to chronic NO synthase inhibition with L‐NAME (Pollock and Rekito 1998). Our previous observation of greater NO bioavailability and blood pressure dependence in the Harlan SD rats could not be recapitulated in the FMD model. Even though NO may play an enhanced role in the maintenance of baseline blood pressure in the Harlan animals, the lack of a FMD difference suggests that blood pressure and FMD responses are unrelated in SD rats. This could mean that peripheral reactive hyperemia in SD animals evokes distinct mechanisms from chronic blood pressure control systems.

Animals from both strains had distinct and robust premyogenic hemodynamic responses with correspondingly prolonged postmyogenic responses. This pattern held true regardless of distributor and supports the theory of shear‐stress‐induced vasodilation in the postmyogenic phase. Flow‐mediated shear stress has been repeatedly identified as the major physiological modulator of vascular tone both in the microcirculation and the conduit arteries (Celermajer et al. 1992; Paniagua et al. 2001). We confirmed this phenomenon and add that shear‐dependent hyperemic responses (postmyogenic flow) are proportional to the initiating shear stimulus (premyogenic flow) in the femoral arteries of rats.

We were surprised at the variability in baseline parameters considering the similarity in age. To determine if these factors influenced reactive hyperemic response, we plotted each factor against the area under the curve reactive hyperemia response. Neither body mass nor baseline femoral artery blood flow had significant linear correlation, whereas there was moderate correlation based on mean arterial pressure at baseline, which was depended on rat strain. Interestingly, in WKY but not SD animals, this seems to reflect prevailing dogma in the literature that illustrates an indirect relationship between arterial pressure and shear‐mediated blood flow (Linder et al. 1990; Panza et al. 1990; Palmieri et al. 2011). This held true for WKY rats in our model of shear‐induced flow where increasing blood pressure resulted in decreased reactive hyperemic responses.

Our experimental procedure was designed to mimic the FMD protocol that is used clinically; however, slight variations exist. In the clinical setting, an arm cuff is typically placed on the forearm and inflated for 5 min, occluding both arterial and venous flow. In our method, the femoral artery was selectively occluded and the differences between this and the approach in humans are thought to be minimal but remain unclear at this point. Additionally, ultrasonographic Doppler technology is used to determine blood flow velocity and changes in artery diameter in the clinic, whereas our procedure was able to more precisely and accurately quantitate actual blood flow through the vessel. This is an advantage over the noninvasive version because we can quantify a functional response to an ischemic stimulus.

In conclusion, rat strain is a major determining factor in NO bioavailability as assessed via femoral artery clamping and subsequent reactive hyperemia. These data have broad ranging implications for cardiovascular research using these animal models, especially when integrating data across different studies or in the setting of a meta‐analysis. Moreover, strain choice is an important factor in the overall outcome of cardiovascular research given genetic dispositions of certain animal strains and should inform future research aimed at measuring these parameters.

## Conflicts of Interest

 No conflicts of interest, financial or otherwise, are declared by the authors.
